# Administration of mesenchymal stem cells in diabetic kidney disease: a systematic review and meta-analysis

**DOI:** 10.1186/s13287-020-02108-5

**Published:** 2021-01-07

**Authors:** Wenshan Lin, Hong-Yan Li, Qian Yang, Guangyong Chen, Shujun Lin, Chunling Liao, Tianbiao Zhou

**Affiliations:** 1 Department of Nephrology, the Second Affiliated Hospital of Shantou University Medical College, No. 69 Dongsha Road, Shantou, 515041 China; 2 Department of Nephrology, Huadu District People’s Hospital of Guangzhou, Southern Medical University, Guangzhou, China

**Keywords:** Mesenchymal stem cell, Diabetic kidney disease, Animal study, Clinical trial, Meta-analysis, Systematic review

## Abstract

**Background:**

Mesenchymal stem cell (MSC) therapy shows great promise for diabetic kidney disease (DKD) patients. Research has been carried out on this topic in recent years. The main goals of this paper are to evaluate the therapeutic effects of MSCs on DKD through a meta-analysis and address the mechanism through a systematic review of the literature.

**Method:**

An electronic search of the Embase, Cochrane Library, ISI Web of Science, PubMed, and US National Library of Medicine (NLM) databases was performed for all articles about MSC therapy for DKD, without species limitations, up to January 2020. Data were pooled for analysis with Stata SE 12.

**Result:**

The MSC-treated group showed a large and statistically significant hypoglycemic effect at 1 week, 2 weeks, 3 weeks, 1 month, 2 months, 3 months, and 6 months. Total hypoglycemic effect was observed (SMD = − 1.954, 95%CI − 2.389 to − 1.519, *p* < 0.001; *I*^2^ = 85.1%). The overall effects on serum creatinine (SCr) and blood urea nitrogen (BUN) were analyzed, suggesting that MSC decreased SCr and BUN and mitigated the impairment of renal function (SCr: SMD = − 4.838, 95%CI − 6.789 to − 2.887, *p* < 0.001; *I*^2^ = 90.8%; BUN: SMD = − 4.912, 95%CI − 6.402 to − 3.422, *p* < 0.001; *I*^2^ = 89.3%). Furthermore, MSC therapy decreased the excretion of urinary albumin. Fibrosis indicators were assessed, and the results showed that transforming growth factor-β, collagen I, fibronectin, and α-smooth muscle actin were significantly decreased in the MSC-treated group compared to the control group.

**Conclusion:**

MSCs might improve glycemic control and reduce SCr, BUN, and urinary protein. MSCs can also alleviate renal fibrosis. MSC therapy might be a potential treatment for DKD.

## Introduction

Diabetes mellitus (DM) is a chronic metabolic disease with a rising incidence rate, and its microvascular and macrovascular complications are associated with a large global burden of morbidity and mortality [1]. Diabetic kidney disease (DKD) is a serious kidney-related complication that is present in approximately 40% of patients with DM [2], and patients with DKD have an increased risk of cardiovascular events and all-cause mortality [3]. Abnormal blood glucose status leads to oxidative stress and induces the release of inflammatory mediators, resulting in glomerular lesions in DM patients. The current evidence indicates that it is very difficult to prevent the progression of DKD. The creatinine clearance rate (CCr), serum creatinine (SCr), blood urea nitrogen (BUN), microalbuminuria, urinary albumin excretion, etc. are important indicators to assess the renal damage associated with DKD.

Mesenchymal stem cells (MSCs) are being used systemically or locally to treat many diseases, as they exhibit great self-renewal and differentiation potential [4, 5]. Stem cells are self-renewing, self-replicating pluripotent cells and can be classified according to their origin: embryonic stem cells, adult stem cells, and induced pluripotent stem cells. Among them, adult stem cells, the undifferentiated cells in differentiated tissues, can be isolated from the bone marrow, adipose tissue, umbilical cord blood, and deciduous teeth. MSCs have been used for tissue regeneration and repair [6], treatment of inflammatory disease [7], prevention of transplant rejection [8], and other clinical applications.

At present, there are some data indicating that MSCs might improve complications from DM [9–11]. We conducted this systematic review and meta-analysis to evaluate the effects of MSC therapy on DKD.

### Search strategy

We searched the Embase, Cochrane Library, ISI Web of Science, PubMed, and US National Library of Medicine (NLM) databases through January 2020 for original papers that assessed the effects of MSC administration on DKD animal models or patients without language restrictions. Keywords in this research included the following: (mesenchymal stem cells OR MSC OR multipotent stromal cells OR mesenchymal stromal cells OR mesenchymal progenitor cells OR Wharton jelly cells OR adipose-derived mesenchymal stem cells OR bone marrow stromal stem cells) AND (diabetic nephropathy OR DN OR diabetic kidney disease OR DKD).

Randomized controlled trials, comparative studies, or controlled trials that assessed the efficacy or safety of MSC therapy for treatment as an intervention in DKD animal models (without species limitations) or patients with DKD were included. The included studies were required to contain biochemical data on renal function or adverse events and to report albuminuria and impaired renal function in patients or animals with DM. The precise distinction between DKD and diabetic nephropathy (DN) was outside the scope of this paper, and both were included. Reviews, case reports, meta-analyses, comments, and letters were excluded. Articles that studied embryonic stem cells, induced pluripotent stem cells, or MSC components (rather than actual MSCs) for the treatment of DKD were excluded. In addition, studies that lacked a control arm or did not provide essential data such as renal function and sample size were excluded. We also searched for additional relevant reports by browsing the references of the articles.

### Data extraction

The main features of the included studies were summarized, and the data were extracted independently by two authors using a standardized datasheet. Adverse events and biochemical indicator data were extracted from the articles, such as blood glucose, CCr, SCr, BUN, U-albumin/U-creatinine ratio (U-ACR), microalbuminuria, urinary albumin excretion, urine protein/Cr, kidney weight, body weight, and kidney weight/body weight ratio. If a paper contained no specific information, data were obtained by measuring the chart in the paper or by contacting the primary authors. Any disagreements in the extracted data were resolved by the third author.

### Validity and quality assessment

For clinical trials, quality assessment was performed using 4 items based on the Jadad scale [12]: randomization, concealment of allocation, blinding method, and description of withdrawals and dropouts. A total score of ≥ 3 was considered high quality.

For animal studies, the methodological quality assessment was carried out using a risk of bias (RoB) tool by the Systematic Review Centre for Laboratory Animal Experimentation (SYRCLE), which is based on the Cochrane RoB tool and adjusted for animal experiments. The following ten items were assessed. (1) Sequence generation: Were the subjects randomly assigned to the case or control groups with an adequately generated allocation sequence? (2) Baseline characteristics: Were the baseline characteristics of the two groups comparable? (3) Allocation concealment: Was the allocation of all the subjects adequately concealed? (4) Random housing: Were all the subjects randomly housed in the same environment during the experiment? (5) Researcher blinding: Were the researchers blinded to which subjects had received treatment (in this case, MSC treatment)? (6) Random outcome assessment: Were the animals selected in random order for outcome assessment? (7) Blinding of outcome assessors: Were the outcome assessors blinded to the group information? (8) Incomplete outcome data: Were incomplete outcome data or dropouts adequately addressed? (9) Selective outcome reporting: Was the study free of selective outcome reporting for significant results? (10) Other sources of bias: Was the study apparently free of other problems that could result in a high risk of bias, such as contamination of MSCs, inappropriate influence of the funder, errors in units of analysis, design-specific risk of bias, and additional animals to replace dropouts? An answer of “yes” means a low risk of bias, while “no” means a high risk of bias, and “unclear” means the risk of bias cannot be assessed for the lack of sufficient information. Disagreements were resolved by consensus-oriented discussion.

### Statistical analysis

Stata SE 12 was used for statistical analysis. For continuous variables, standard mean differences (SMDs) were obtained by pooling the mean values, standard deviations, and sample sizes. For binary data, the odds ratio (OR) was calculated. Moreover, 95% confidence intervals (95%CIs) were calculated between the MSC-treated groups and the control groups. If there were multiple MSC-treated groups in an article, the data in the control group were reused. Heterogeneity across studies was quantified using *I*^2^ and was considered significant at a *p* value of < 0.1. The data were pooled using a fixed-effect model without heterogeneity or a random-effect model. A *p* value of < 0.05 was regarded as statistically significant for all analyses. Potential publication bias was assessed with Begg’s test, Egger’s test, and the trim-and-fill method.

## Results

### Search results

In total, 33 studies in 29 publications were included, among which 28 publications were based on animal studies [13–40] and 1 was based on a clinical trial [41]. In addition, there are 4 ongoing clinical trials registered with the NLM.

Among 32 animal studies, 24 studies used rat models, 7 used mouse models, and 1 used a rhesus macaque model. A single method or a combination of multiple methods was used to induce DM, including streptozotocin (STZ) injection, high-fat diet dietary induction, nephrectomy, and natural development of models. However, the dosage and frequency of STZ injection and the time when the animals were tested for the establishment of DN were different. Although MSCs were used in all the included studies, the details of the source, dosage, frequency, administration, and point in time varied. The sources of MSCs were bone marrow mesenchymal stem cells (BM-MSCs) in 22 studies, adipose-derived stem cells (ADSCs) in 4 studies, human umbilical cord blood-derived mesenchymal stem cells (hUCB-MSCs) in 5 studies, and stem cells from exfoliated deciduous teeth in 1 study. Allogeneic administration was used in 23 studies, xenoplastic administration was used in 8 studies, and autologous administration was used in 1 study. The characteristics of the included animal studies are summarized in Table 1.

**Table 1:** Main features of included studies (animal studies)

	PMID	Year	Author	Country	Sample size	Model features	Comparison	Stem cell species	Intervention	Observed indicators	Duration
1	18,489,988	2008	Ezquer et al.	Chile	16	STZ was injected intraperitoneally at a dose of 40 mg/kg, for 5 consecutive days.	STZ + MSCs vs STZ + vehicle	C57BL/6 mouse bone marrow (allogeneic transplantation)	Twenty-five days after the first STZ dose, mice received 0.5 × 10^6^ MSCs or the vehicle via the tail vein.	Blood glucose; urinary glucose; intraperitoneal glucose tolerance test; urine albumin/creatinine ratio; pancreas and kidney histopathology	62 days
2	19,822,294	2009	Ezquer et al.	Chile	16	C57BL/6 mice received intraperitoneally 200 mg/kg STZ.	STZ + MSCs vs STZ + vehicle	C57BL/6 mouse bone marrow (allogeneic transplantation)	Thirty and 51 days after STZ injection, animals received via the tail vein the vehicle (untreated) or 0.5 × 10^6^ MSC (MSC treated).	Blood glucose; urinary glucose; insulinemia; serum creatinine; urine albumin/creatinine ratio; pancreas and kidney histopathology; kidney weight/body mass ratio	90 days
3	19,951,572	2009	Zhou et al.	China	32	The male rats received a single intraperitoneal injection of STZ (60 mg/kg).	STZ + MSCs vs STZ + vehicle	SD rat bone marrow (allogeneic transplantation)	2 × 10^6^ labeled MSCs per animal in 0.2 mL SFM were given via the left cardiac ventricle. Control diabetic animals were treated identically but infused with 0.2 mL SFM instead of cells.	Blood glucose; kidney weight/body mass ratio; urine albumin/creatinine ratio; blood pressure; creatinine clearance rate; kidney histopathology	2 months
4	20,067,112	2009	Zhou et al.	China	24	A single intraperitoneal injection of STZ (60 mg/kg) was given to SD rats.	STZ + MSCs vs STZ + vehicle	SD rat bone marrow (allogeneic transplantation)	2 × 10^6^/200 μL MSCs were given via the left cardiac ventricle. A week later, the second intracardiac injection of the MSCs was performed. Control diabetic animals received 200 μL serum-free DMEM-LG.	Blood glucose; body mass; urine protein; kidney/body mass ratio; creatinine clearance rate; kidney histopathology	2 months
5	22,552,764	2012	Fang et al.	China	24	Rats were injected intraperitoneally with 40 mg/kg body weight of STZ for 5 consecutive days.	STZ + MSCs vs STZ + vehicle	SD rat adipose tissue (autologous transplantation)	Intravenous infusion of autologous ADMSCs (1.0 × 10^7^) was performed 4 weeks after the onset diabetes via the tail vein. Animals in the vehicle group received an equal volume of culture medium at the same time.	Blood glucose; insulinemia; cholesterol; triglycerides; BUN; creatinine; malondialdehyde; TNF-α; IL-1β; IL-6; renal morphology	12 weeks
6	22,564,642	2012	Park et al.	Korea	14	Experimental diabetes was induced by intravenous injection of STZ (50 mg/kg).	STZ + MSCs vs STZ + vehicle	Human umbilical cord blood (xenoplastic transplantation)	hUCB-SC (1 × 10^6^ cells/rat) were infused through the tail vein 4 weeks after the STZ injection. Both diabetic and diabetic rats treated with hUCB-SC were injected subcutaneously with insulin (2 U/day/rat) to maintain blood glucose levels of 350 to 500 mg/dL.	Blood glucose; body mass; kidney weight; creatinine; urinary protein; fibronectin; α-SMA; E-cadherin	1 month
7	23,026,513	2012	Park et al.	Korea	14	Experimental diabetes was induced by injecting 50 mg/kg STZ through the tail vein.	STZ + MSCs vs STZ + vehicle	Human umbilical cord blood (xenoplastic transplantation)	hUCB-MSC were infused at a dose of 5 × 10^5^ cells/rat through the tail vein 2 days after the STZ injection when blood glucose was > 350 mg/dL.	Blood glucose; body mass; kidney weight; creatinine; urinary protein; kidney histopathology; BMP-7; TGF-β1; fibronectin; α-SMA; E-cadherin	1 month
8	23,295,166	2013	Wang et al.	China	17	Diabetes was induced by a single intraperitoneal injection of streptozotocin (STZ) (65 mg/kg) in the rats following overnight fasting.	STZ + MSCs vs STZ + vehicle	SD rat bone marrow (allogeneic transplantation)	MSC-treated DN rats were injected with 2 × 10^6^ MSC via the left renal artery. All the diabetic rats received daily injections of insulin to maintain blood glucose levels between 16 and 28 mmol/L.	Blood glucose; body mass; kidney weight; kidney/body mass ratio; creatinine clearance rate; urine albumin/creatinine ratio; creatinine; renal morphology; nephrin; podocin; VEGF; BMP-7	2 months
9	23,762,850	2013	Zhang et al.	China	20	SD rats were anesthetized and received a single intraperitoneal injection of 60 mg/kg STZ.	STZ + MSCs vs STZ + vehicle	SD rat bone marrow (allogeneic transplantation)	MSCs (1 × 10^6^) were resuspended in 2 mL of PBS and administrated to anesthetized rats through tail vein.	Blood glucose; insulinemia; urine albumin/creatinine ratio; kidney and pancreas histopathology; VCAM-1; TGF-β1; IL-10; synaptopodin	8 weeks
10	23,791,972	2013	Lv et al.	China	32	Diabetes was induced in the female Wistar rats by a single intraperitoneal injection of STZ (60 mg/kg) after one-night fasting.	STZ + MSCs vs STZ + vehicle	Wistar rat bone marrow (allogeneic transplantation)	MSCs were transplanted via the tail vein at a concentration of 2 × 10^6^ in 0.5 mL serum-free media once a week for 2 continuous weeks.	Blood glucose; urinary albumin excretion; creatinine clearance rate; kidney/body mass ratio; kidney histopathology; ED-1; MCP-1; collagen I; fibronectin; IL-1β; IL-6; TNFα; HGF	8 weeks
11	24,513,119	2013	Lv et al.	China	24	Following one night of fasting, a single injection of STZ (60 mg/kg) was given intraperitoneally to induce diabetes.	STZ + MSCs vs STZ + vehicle	Wistar rat bone marrow (allogeneic transplantation)	MSCs were transplanted via the tail vein at a concentration of 2 × 10^6^ in 0.5 mL serum-free media once a week for 2 continuous weeks.	Blood glucose; urinary albumin excretion; creatinine clearance rate; kidney/body mass ratio; renal histopathology; collagen I; FN; TGF-β; MDA; SOD; ROS	8 weeks
12	24,606,996	2014	Abdel Aziz et al.	Egypt	40	Diabetes of female albino rats was induced by a single intraperitoneal injection of STZ (60 mg/kg body weight).	STZ + MSCs vs STZ + vehicle	White albino rat bone marrow (allogeneic transplantation)	DN rats received MSCs in a single dose of 10^6^ cells per rat by intravenous injection in the rat tail vein.	Blood glucose; BUN; creatinine; urinary albumin excretion; body weight; renal histopathology; TGF β; TNFα; bcl2; Bax; VEGF	4 weeks
13	24,845,071	2015	Lv et al.	China	24	A single injection of STZ (60 mg/kg) was given via intraperitoneal injection to induce diabetes following one-night fasting.	STZ + MSCs vs STZ + vehicle	Wistar rat bone marrow (allogeneic transplantation)	MSCs were transplanted via the tail vein at a concentration of 2 × 10^6^ in 0.5 mL serum-free media once a week for two continuous weeks.	Blood glucose; urinary albumin excretion; creatinine clearance rate; kidney/body mass ratio; renal histopathology; TGF-β; collagen I; collagen IV; α-SMA; E-cadherin; BMP7; Smad2; Smad3	8 weeks
14	27,018,336	2016	Lang et al.	China	20	After fasting 12 h, SD rats were given STZ 55 mg/kg by i.p. injection.	STZ + MSCs vs STZ + vehicle	SD rat bone marrow (allogeneic transplantation)	For rats in the MSC group, intravenous infusion of autologous MSCs (2 × 10^6^/mL) was performed 4 weeks after the onset of diabetes via the tail vein.	Blood glucose; body weight; urinary protein; creatinine; renal mass index; kidney histopathology; MMP- 9; PAI-1; TGF-β1; Smad3	12 weeks
15	27,721,418	2016	Nagaishi et al.	Japan	12	Diabetes was induced via an HFD containing 60% lard (high-fat diet 32) for 28 weeks.	HFD + MSCs vs HFD + vehicle	C57BL/6-GFP-transgenic mouse bone marrow (allogeneic transplantation)	C57BL/6J mice were administered 1.0 × 10^4^ MSCs/g body weight 4 times (HFD-MSC) every 2 weeks.	Blood glucose; urine albumin/creatinine ratio; kidney histopathology; ICAM-1; TNF-α; megalin; TGF-β; ZO-1	8 weeks
16	27,721,418	2016	Nagaishi et al.	Japan	12	Diabetes was induced by a single intraperitoneal injection of STZ (150 mg/kg).	STZ + MSCs vs STZ + vehicle	C57BL/6-GFP-transgenic mouse bone marrow (allogeneic transplantation)	C57BL/6J mice were administered 1.0 × 10^4^ MSCs/g body weight 2 times (STZ-MSC) every 4 weeks.	Blood glucose; urine albumin/creatinine ratio; kidney histopathology; ICAM-1; TNF-α; megalin; TGF-β; ZO-1	8 weeks
17	27,774,826	2016	Hamza et al.	Egypt	20	Albino Wistar rats were given a single intraperitoneal injection of a mixture of 70 mg/kg STZ.	STZ + MSCs vs STZ	Albino rat bone marrow (allogeneic transplantation)	Rats were given a single-dose intravenous treatment of 1.0 × 10^6^ cells per subject.	Blood glucose; insulinemia; BUN; creatinine; uric acid; serum total protein; serum albumin; urinary urea, urinary creatinine; microalbumin; kidney histopathology; HO-1; AGEP; FGF; PDGF; TGF-β; IL-8; MCP-1	3 weeks
18	28,814,814	2016	Nagaishi et al.	Japan	8	C57BL/6 mice were given a single intraperitoneal administration high dose (150 mg/kg) of STZ.	STZ + MSCs vs STZ + vehicle	SD rat bone marrow (xenoplastic transplantation)	Mice were administered 4 times with 1 × 10^4^ MSCs/g body weight via the tail vein every 2 weeks.	Blood glucose; urine albumin/creatinine ratio; kidney histopathology	8 weeks
19	28,814,814	2016	Nagaishi et al.	Japan	10	SD rats were given a single tail vein injection of 55 mg/kg of STZ.	STZ + MSCs vs STZ + vehicle	SD rat bone marrow (allogeneic transplantation)	Rats were administered 1 × 10^4^ MSCs/g body weight via the tail vein.	Blood glucose; urine albumin/creatinine ratio; kidney histopathology	8 weeks
20	28,814,814	2016	Nagaishi et al.	Japan	16	OLETF rats developed diabetes and DN with natural course.	OLETF + MSCs vs OLETF + vehicle	LETF rat bone marrow (allogeneic transplantation)	Rats were administered 1 × 10^4^ MSCs/g body weight via the tail vein.	Blood glucose; urine albumin/creatinine ratio; kidney histopathology	8 weeks
21	29,425,466	2018	Rashed et al.	Egypt	20	Diabetes was induced by a single intraperitoneal injection of STZ (50 mg/kg).	STZ + vehicle vs STZ + MSCs	Wistar strain Albino rat bone marrow (allogeneic transplantation)	DN rats treated with a single injection of 1 × 10^6^ labeled MSCs per animal in 0.5 mL serum-free medium into the tail vein.	Blood glucose; insulinemia; BUN; creatinine; creatinine clearance rate; urinary albumin excretion; kidney histopathology; TNF-α; IL-10; SOD; TGF-β; Beclin-1	2 weeks
22	29,484,379	2018	Li et al.	China	25	Diabetes was induced by a single intraperitoneal injection of 55 mg/kg STZ.	STZ + MSCs vs STZ + vehicle	SD rat bone marrow (allogeneic transplantation)	2, 4, 5, and 7 weeks after successful establishment of the diabetes model, MSCs were transplanted via the tail vein at a concentration of 5 × 10^6^ cells.	Microalbumin; urine albumin/creatinine ratio; BUN; kidney histopathology; MCP-1; IL-1β; TNF- α; ICAM-1; CD68; TGF- β; fibronectin; IL-1 α; IL-2; IL-6; EGF; IL-10; TNF- α; IFN- γ; GRO; VEGF	8 weeks
23	31,023,998	2019	Bai et al.	China	24	Diabetes was induced by a single intraperitoneal injection of 60 mg/kg STZ after 1-night fasting.	STZ + MSCs vs STZ + vehicle	SD rat bone marrow (allogeneic transplantation)	MSCs were transplanted via the tail vein at a concentration of 5 × 10^6^ in 0.5 mL PBS once a week for 2 continuous weeks.	Blood glucose; creatinine; BUN; urinary glucose; microalbumin; albumin/creatinine ratio; kidney histopathology; TGF-β; Smad2/3; phosphorylated Smad2; phosphorylated Smad3; IFN- γ; IL-8; IL-6; TNF-α	12 weeks
24	31,150,720	2019	Xian et al.	China	19	Healthy female NOD mice were purchased. When two consecutive tests showed a blood glucose level > 16.6 mmol/L, the mouse was diagnosed with T1DM.	NOD-T1DM + MSCs vs NOD-T1DM	Human umbilical cord-derived MSCs (xenoplastic transplantation)	1 × 10^6^ hUCMSCs suspended in 0.3 mL of phosphate-buffered saline (PBS) were injected into the tail vein on the 3rd day after diabetes onset (only once).	Blood glucose; weight; creatinine; BUN; urinary albumin excretion; MCP-1; RAGE; nephrin; WT1; NF-κB	8 weeks
25	31,190,436	2019	Cai et al.	China	20	Wistar rats were provided with a prepared HFD for 6 weeks and then injected with 60 mg/kg STZ for 2 weeks.	HFD + STZ + MSCs vs HFD + STZ + vehicle	Rat bone marrow stromal cells (allogeneic transplantation)	Rats treated with 2 × 10^6^ labeled MSCs per animal via the tail vein.	Blood glucose; creatinine; BUN; alanine aminotransferase; 24 h urine volume; urine protein; urinary albumin excretion; renal histopathology; p-cadherin; synaptopodin; FSP-1; α-SMA; snail; fibronectin; collagen I	12 weeks
26	31,285,429	2019	Lee et al.	Korea	14	CD1 mice were intraperitoneally injected with STZ 80 mg/kg for 3 days.	STZ + MSCs vs STZ + vehicle	Human umbilical cord-derived MSCs (xenoplastic transplantation)	Five weeks after the induction of diabetes, human umbilical cord blood-derived MSCs were administered to mice three times.	Blood glucose; creatinine; BUN; urine albumin/creatinine ratio	19 weeks
27	31,622,047	2019	Takemura et al.	Japan	10	SDT fatty rats formed a spontaneously obese type 2 diabetes model and were subjected to right nephrectomy.	Nephrectomy + MSCs vs nephrectomy	EGFP rat subcutaneous adipose tissue (allogeneic transplantation)	1 mL of the adipose-derived mesenchymal stem cell suspension (6.0 × 10^6^ cells/mL) was administered via the femoral vein.	Albuminuria; proteinuria; urinary creatinine; podocalyxin; L-FABP; KIM-1; TNF-α; IL-6; renal histopathology	2 weeks
28	31,622,047	2019	Takemura et al.	Japan	11	SDT fatty rats formed a spontaneously obese type 2 diabetes model and were subjected to right nephrectomy.	Nephrectomy + MSCs vs nephrectomy	EGFP rat subcutaneous adipose tissue (allogeneic transplantation)	Adipose-derived mesenchymal stem cell sheets were laminated in three layers under the renal capsule using a cell sheet transfer device.	Albuminuria; proteinuria; urinary creatinine; podocalyxin; L-FABP; KIM-1; TNF-α; IL-6; renal histopathology	2 weeks
29	31,747,961	2019	Yu et al.	China	12	SD rats were fed a HFD for 8 weeks, followed by an STZ injection at a single dose of 25 mg/kg. HFD feeding was maintained in the newly diabetic rats for 24 weeks.	HFD + STZ + MSCs vs HFD + STZ + vehicle	SD rat adipose tissue (allogeneic transplantation)	Rats were treated through the tail vein with a single infusion of 3 × 10^6^ ADSCs once a week for 24 weeks.	Blood glucose; creatinine; BUN; urine albumin/creatinine ratio; ALT; AST; ALP; LDL-C; TC; TG; renal histopathology; insulin; glucagon; collagen I; α-SMA; CD163; albumin; SP-C; CD206; PI3K; p-AKT; IL-1β; IL-6; IL-10; TNF-α	25 weeks
30	31,791,397	2019	An et al.	China	12	Rhesus macaques were administered a single high dose of STZ (80 mg/kg) intravenously. Insulin was used to maintain the FBG level at 15–20 mmol/L.	STZ + MSCs vs STZ + vehicle	Human umbilical cord-derived MSCs (xenoplastic transplantation)	MSCs from a single donor were suspended in 100 mL normal saline and delivered at a density of 2 × 10^6^ cells/kg to one DN rhesus macaque at an infusion rate of 45–50 drops/min. A total of four times of MSC transplantation were performed during 2 months.	Blood glucose; serum creatinine; BUN; uric acid; LDL-C; HDL-C; TC; TG; HbA1c; eGFR; microalbumin; urinary creatinine; urine albumin/creatinine ratio; body weight; renal histopathology; IL-1β; IL-16; IL-8; IL-6; IL-10; TNF-α; TGF-β; CCL-5; SGLT-2	1 year
31	31,871,464	2019	Rao et al.	China	18	GK rats were given a HFD for 2–4 weeks.	HFD + MSCs vs HFD + vehicle	Human bone marrow MSCs from donors aged 16–20 (xenoplastic transplantation)	A total of 4 × 10^6^ MSC cells were administered via the tail vein to each rat.	Blood glucose; body weight; serum cholesterol; serum triglycerides; urinary albumin; kidney/body mass ratio; renal histopathology; α-SMA; Col I; fibronectin; lamininβ; nephrin; synaptopodin; IL-1β; IL-6; IL-10; TNF-α; TGF-β; HGF	8 weeks
32	31,871,464	2019	Rao et al.	China	20	GK rats were given HFD for 2–4 weeks.	HFD + MSCs vs HFD + vehicle	Human exfoliated deciduous tooth stem cells from donors aged 6–8 (xenoplastic transplantation)	A total of 4 × 10^6^ MSC cells were administered via the tail vein to each rat.	Blood glucose; body weight; serum cholesterol; serum triglycerides; urinary albumin; kidney/body mass ratio; renal histopathology; α-SMA; Col I; fibronectin; lamininβ; nephrin; synaptopodin; IL-1β; IL-6; IL-10; TNF-α; TGF-β; HGF	8 weeks

*SD* Sprague-Dawley, *SFM* serum-free medium, *HFD* high-fat diet, *BUN* blood urea nitrogen, *NOD* non-obesity diabetes, *OLETF* Otsuka Long-Evans Tokushima Fatty, *LETO* Long-Evans Tokushima Otsuka, *MDA* malondialdehyde, *SOD* superoxide dismutase, *ICAM-1* intracellular adhesion molecule-1, *ZO-1* zona occludens protein-1, *HO-1* heme-oxygenase-1, *AGEP* advanced glycation end product, *FGF* fibroblast growth factor, *PDGF* platelet-derived growth factor, *EGF* epidermal growth factor, *RAGE* advanced glycation end products, *FSP-1* fibroblast-specific protein-1, *L-FABP* liver-type fatty acid-binding protein, *KIM-1* kidney injury molecule-1, *ALT* alanine aminotransferase, *AST* aspartate aminotransferase, *ALP* alkaline phosphatase, *LDL-C* low-density lipoprotein cholesterol, *TC* total cholesterol, *TG* triglyceride, *SP-C* pro-surfactant protein C, *eGFR* estimated glomerular filtration rate, *SGLT-2* Na + -glucose cotransporter 2

The only clinical trial was a multicenter, randomized, double-blind, dose-escalating, sequential, placebo-controlled study, finished in 2016. Thirty patients were randomized to receive one of two doses of mesenchymal precursor cells or placebo, and the efficacy and adverse events were observed. The main features of the clinical trial are shown in Table 2.

**Table 2:** Main features of included studies (clinical trials)

Author, year	Study design	Treatment strategies	Detailed scheme	Patient characteristics	Main outcome measures	Adverse events	Study duration
Packham et al., 2016	Multicenter, randomized, double-blind, dose-escalating, sequential, placebo-controlled trial	Vehicle vs MPC	Two rexlemestrocel-L (allogeneic mesenchymal precursor cells) doses (150 × 10^6^ or 300 × 10^6^) or saline placebo were suspended in 100 mL normal saline and infused with filtration over 45 min.	The study population was male and female patients ≥ 45 and ≤ 85 years old with type 2 diabetes and advanced diabetic nephropathy (e.g., eGFR20—50 mL/min/1.73 m^2^) who were receiving a stable, standard of care therapeutic regimen of the maximum tolerated recommended dose of an angiotensin converting enzyme inhibitor (ACEI) or a angiotensin 2 receptor blocker (ARB) for at least 3 months prior to screening.	Adverse events; serum creatinine; creatinine clearance; albumin-creatinine ratio; protein-creatinine ratio; cystatin-C; HbA1c; triglycerides; systolic blood pressure; diastolic blood pressure; hs-CRP; IL-6; TNF-α	Edema peripheral; lower respiratory tract infection; urinary tract infection; cataract; anemia; fall; acute myocardial infarction; anemia; asthma; cardiac failure congestive; cardiac failure congestive; syncope; upper gastrointestinal hemorrhage; gangrene; infected skin ulcer; atrial fibrillation; renal failure chronic; benign prostatic hyperplasia; diabetic ulcer; diverticulitis	60 weeks

None of the animal experiments reported the occurrence of graft rejection after administration, but 2 MSC-treated human patients developed antibodies specific to the donor HLA in the clinical trial, one of these cases occurred transiently, whereas the other presented at baseline and persisted throughout the observation period without the appearance of adverse events. Strangely, however, antibodies specific to the donor HLA were also found in one placebo-treated patient. Six animal experiments specified the deaths or dropouts. Lang and Dai [27] reported the deaths of 6 model rats during the construction of the diabetes model (21.4%, 6/28), and Wang et al. [21] reported 1 death each in the MSC-treated group (8.3%, 1/14) and the DN group (10%, 1/10) as well as 2 deaths because of anesthesia. In the study of Li et al. [32], no rat died in the DN group (0.0%, 0/14), and 2 died in the MSC-treated group (18.2%, 2/11). During a 12-week observation, the MSC-treated group (25%, 3/12) had lower mortality than the DN-treated group (66.7%, 8/12) [33]. Similarly, Xian et al. [34] found 2 deaths in the hUCB-MSC group (16.7%, 2/12), making for a markedly lower mortality rate than the T1DM group (40%, 6/15) at the end of the study. An et al. [39] found no marked change in the immune system of rhesus macaque DN models in response to hUCB-MSC treatment.

### Quality assessment

Quality assessments of animal experiments and clinical trials were performed (Tables 3 and 4). Table 3 shows a number of “unclear” judgments in the quality assessment of animal experiments; in particular, outcome assessment in a random order, concealment of allocation and blinding of outcome assessors in all included experiments were rated “unclear,” largely due to a lack of awareness of randomization and blinding methods in animal experiments. As shown in Table 4, a total score of 7 suggested the high methodological quality of the included clinical trial.

**Table 3:** Quality assessment of animal intervention studies by SYRCLE

	Study	PMID	Sequence generation	Baseline characteristics	Allocation concealment	Random housing	Researchers blinding	Random outcome assessment	Outcome assessors blinding	Complete outcome data	Outcome reporting	Other source of bias
1	Ezquer, 2008	18,489,988	Yes	Yes	Unclear	Unclear	Unclear	Unclear	Unclear	Unclear	Yes	Yes
2	Ezquer, 2008	19,822,294	Yes	Yes	Unclear	Unclear	Unclear	Unclear	Unclear	Unclear	Yes	Yes
3	Zhou, 2009	19,951,572	Unclear	Yes	Unclear	Yes	Unclear	Unclear	Unclear	Unclear	Yes	Yes
4	Zhou, 2009	20,067,112	Yes	Unclear	Unclear	Unclear	No	Unclear	Unclear	Unclear	Yes	Yes
5	Fang, 2012	22,552,764	Yes	Unclear	Unclear	Yes	No	Unclear	Unclear	Unclear	Yes	Yes
6	Park, 2012	22,564,642	Yes	Unclear	Unclear	Unclear	No	Unclear	Unclear	Unclear	Yes	Yes
7	Park, 2012	23,026,513	Yes	Unclear	Unclear	Yes	No	Unclear	Unclear	Unclear	Yes	Yes
8	Wang, 2013	23,295,166	Yes	Yes	Unclear	Yes	Unclear	Unclear	Unclear	Yes	Yes	Yes
9	Zhang, 2013	23,762,850	Yes	Yes	Unclear	Yes	No	Unclear	Unclear	Unclear	Yes	Yes
10	Lv, 2013	23,791,972	Yes	Yes	Unclear	Yes	No	Unclear	Unclear	Unclear	Yes	Yes
11	Lv, 2013	24,513,119	Unclear	Unclear	Unclear	Yes	Unclear	Unclear	Unclear	Unclear	Yes	Yes
12	Abdel Aziz, 2014	24,606,996	Unclear	Yes	Unclear	Yes	Unclear	Unclear	Unclear	Unclear	Yes	Yes
13	Lv, 2015	24,845,071	Yes	Yes	Unclear	Yes	Unclear	Unclear	Unclear	Unclear	Yes	Yes
14	Lang, 2016	27,018,336	Yes	Yes	Unclear	Unclear	Unclear	Unclear	Unclear	Yes	Yes	Yes
15	Nagaishi, 2016	27,721,418	No	Yes	Unclear	Yes	Unclear	Unclear	Unclear	Unclear	Yes	Yes
16	Nagaishi, 2016	27,721,418	No	Yes	Unclear	Yes	Unclear	Unclear	Unclear	Unclear	Yes	Yes
17	Hamza, 2016	27,774,826	No	Unclear	Unclear	Unclear	No	Unclear	Unclear	Unclear	Yes	Yes
18	Nagaishi, 2016	28,814,814	Unclear	Yes	Unclear	Unclear	Unclear	Unclear	Unclear	Unclear	Yes	Yes
19	Nagaishi, 2016	28,814,814	Unclear	Yes	Unclear	Unclear	Unclear	Unclear	Unclear	Unclear	Yes	Yes
20	Nagaishi, 2016	28,814,814	Unclear	Yes	Unclear	Unclear	Unclear	Unclear	Unclear	Unclear	Yes	Yes
21	Rashed, 2018	29,425,466	Yes	Yes	Unclear	Yes	Unclear	Unclear	Unclear	Unclear	Yes	Yes
22	Li, 2018	29,484,379	Yes	Yes	Unclear	Yes	Unclear	Unclear	Unclear	Yes	Yes	Yes
23	Bai, 2019	31,023,998	Yes	Yes	Unclear	Unclear	Unclear	Unclear	Unclear	Yes	Yes	Yes
24	Xian, 2019	31,150,720	Yes	Yes	Unclear	Yes	Unclear	Unclear	Unclear	Yes	Yes	Yes
25	Cai, 2019	31,190,436	Yes	Yes	Unclear	Yes	Unclear	Unclear	Unclear	Unclear	Yes	Yes
26	Lee, 2019	31,285,429	Unclear	Unclear	Unclear	Unclear	Unclear	Unclear	Unclear	Unclear	No	Yes
27	Takemura, 2019	31,622,047	Unclear	Unclear	Unclear	Unclear	Unclear	Unclear	Unclear	Unclear	Yes	Yes
28	Takemura, 2019	31,622,047	Unclear	Unclear	Unclear	Unclear	Unclear	Unclear	Unclear	Unclear	Yes	Yes
29	Yu, 2019	31,747,961	Yes	Yes	Unclear	Yes	Yes	Unclear	Unclear	Unclear	Yes	Yes
30	An, 2019	31,791,397	Yes	Yes	Unclear	Yes	Unclear	Unclear	Unclear	Yes	Yes	Yes
31	Rao, 2019	31,871,464	Yes	Yes	Unclear	Yes	Unclear	Unclear	Unclear	Unclear	Yes	Yes
32	Rao, 2019	31,871,464	Yes	Yes	Unclear	Yes	Unclear	Unclear	Unclear	Unclear	Yes	Yes

**Table 4:** Quality assessment of clinical trials by Jadad score

Author, year	Type	Randomization	Concealment of allocation	Double blinding	Withdrawals and dropouts	Jaded score
Packham et al., 2016	Multicenter, randomized, double-blind, dose-escalating, sequential, placebo-controlled trial	An Interactive Voice Response System/Interactive Web Response System (IVRS/IWRS) was accessed to randomize eligible patients.	All infusions were prepared by an unblinded pharmacist at the phase 1 unit who provided to the blinded clinical staff visually identical infusion products comprising rexlemestrocel-L or saline suspended in 100 mL normal saline.	Patients, investigators, and the sponsor were blinded to the treatment allocation through the entire 60-week study.	Yes	7

### Assessment of glucose

**Fig. 1:**
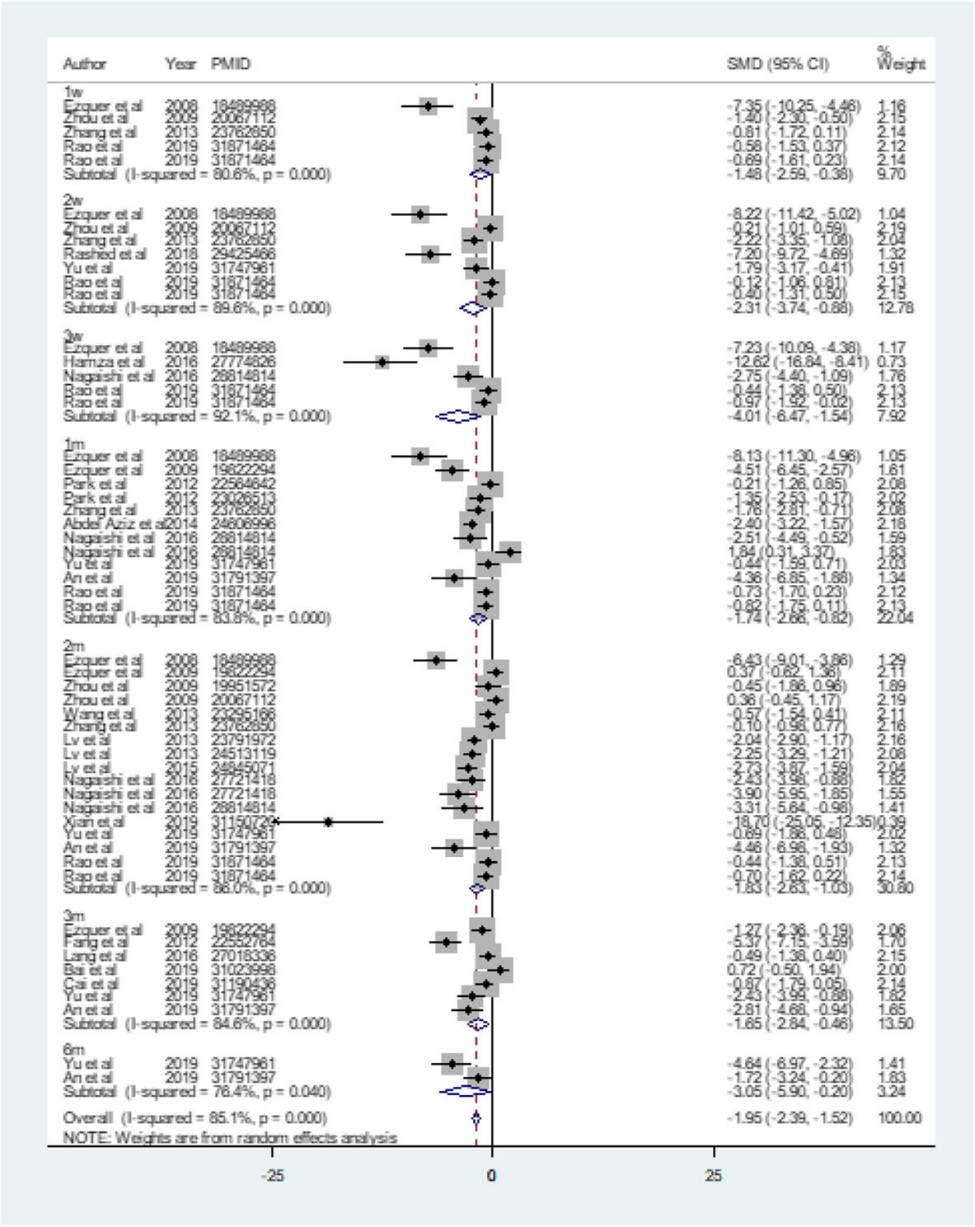
The effect of MSC treatment on glycemic control. 1w, 1 week; 2w, 2 weeks; 3w, 3 weeks; 1m, 1 month; 2m, 2 months; 3m, 3 months; 6m, 6 months

Glucose was detected after MSC treatment in all but 2 studies [32, 37]. Sixteen studies measured glucose once at the end of the experiment [18–21, 23–29, 31, 33–36]. Seven studies conducted blood glucose monitoring at several points in time [14–17, 22, 30, 40]. Five studies, 7 studies, 5 studies, 12 studies, 17 studies, 7 studies, and 2 studies were included to assess the effect of treatment on blood glucose levels at 1 week, 2 weeks, 3 weeks, 1 month, 2 months, 3 months, and 6 months, respectively, all of which showed a highly significant hypoglycemic effect in the MSC-treated group (1 week: SMD = − 1.484, 95%CI − 2.586 to − 0.381, *p* < 0.001; *I*^2^ = 80.6%; 2 weeks: SMD = − 2.312, 95%CI − 3.743 to − 0.882, *p* = 0.002, *I*^2^ = 89.6%; 3 weeks: SMD = − 4.007, 95%CI − 6.472 to − 1.541, *p* = 0.001, *I*^2^ = 92.1%; 1 month: SMD = − 1.740, 95%CI − 2.660 to − 0.821, *p* < 0.001, *I*^2^ = 83.8%; 2 months: SMD = − 1.830, 95%CI − 2.633 to − 1.028, *p* < 0.001; *I*^2^ = 86.0%; 3 months: SMD = − 1.649, 95%CI − 2.838 to − 0.461, *p* = 0.007; *I*^2^ = 84.6%; 6 months: SMD = − 3.045, 95%CI − 5.895 to − 0.195, *p* = 0.036; *I*^2^ = 76.4%). The total hypoglycemic effect was also analyzed (SMD = − 1.954, 95%CI − 2.389 to − 1.519, *p* < 0.001; *I*^2^ = 85.1%) (Fig. 1).

### Assessment of serum creatinine

**Fig. 2:**
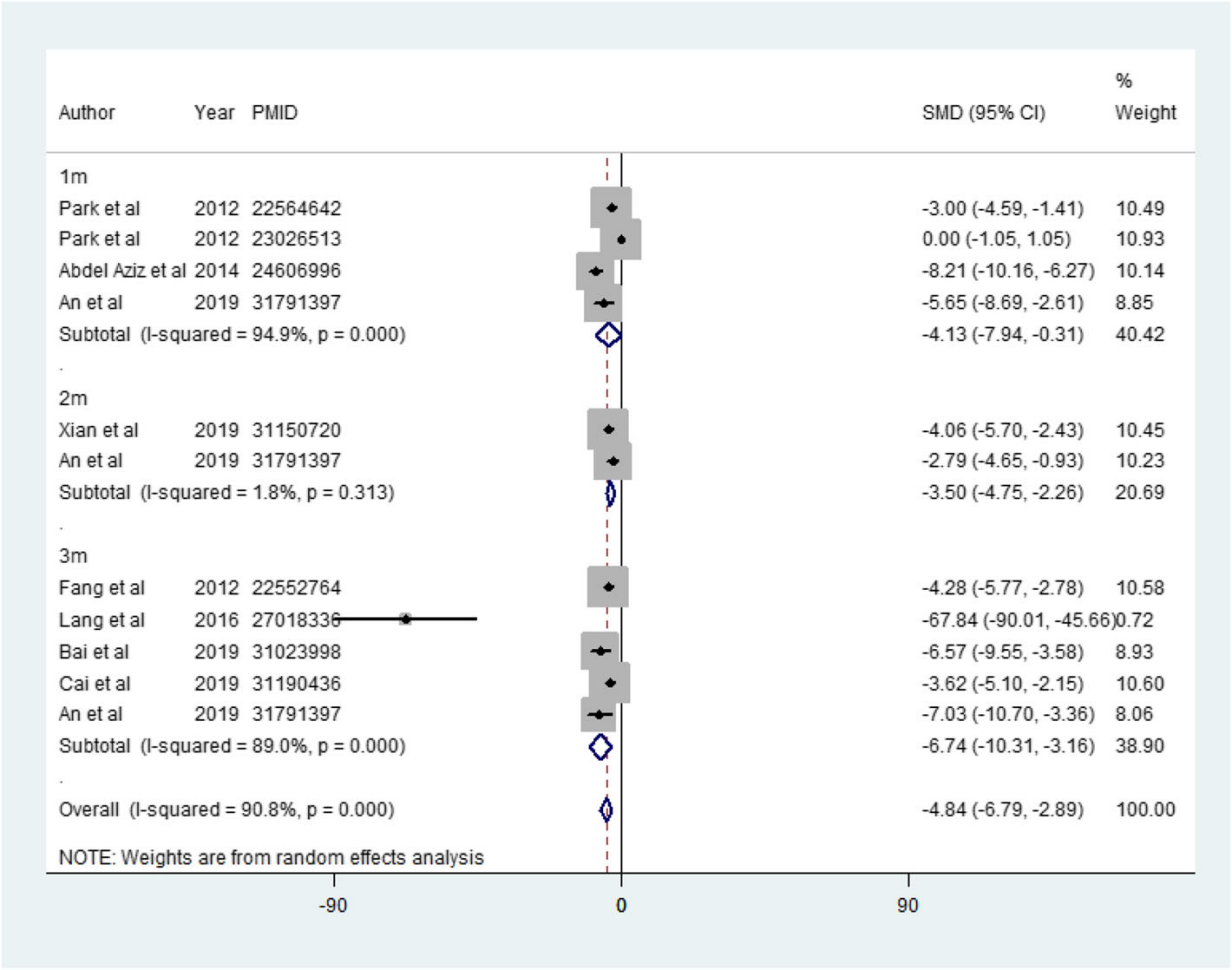
The effect of MSC treatment on serum creatinine. 1m, 1 month; 2m, 2 months; 3m, 3 months

There were 4 studies, 2 studies, and 5 studies that assessed SCr at 1 month, 2 months, and 3 months, respectively. All of them showed significantly reduced creatinine values in the MSC-treated group (1 month: SMD = − 4.126, 95%CI − 7.936 to − 0.315, *p* = 0.034; *I*^2^ = 94.9%; 2 months: SMD = − 3.505, 95%CI − 4.746 to − 2.264, *p* < 0.001; *I*^2^ = 1.8%; 3 months: SMD = − 6.736, 95%CI − 10.311 to − 3.162, *p* < 0.001; *I*^2^ = 89.0%). The total effect on SCr was also analyzed, suggesting that MSCs decreased SCr and improved renal function (SMD = − 4.838, 95%CI − 6.789 to − 2.887, *p* < 0.001; *I*^2^ = 90.8%) (Fig. 2).

### Assessment of blood urea nitrogen

**Fig. 3:**
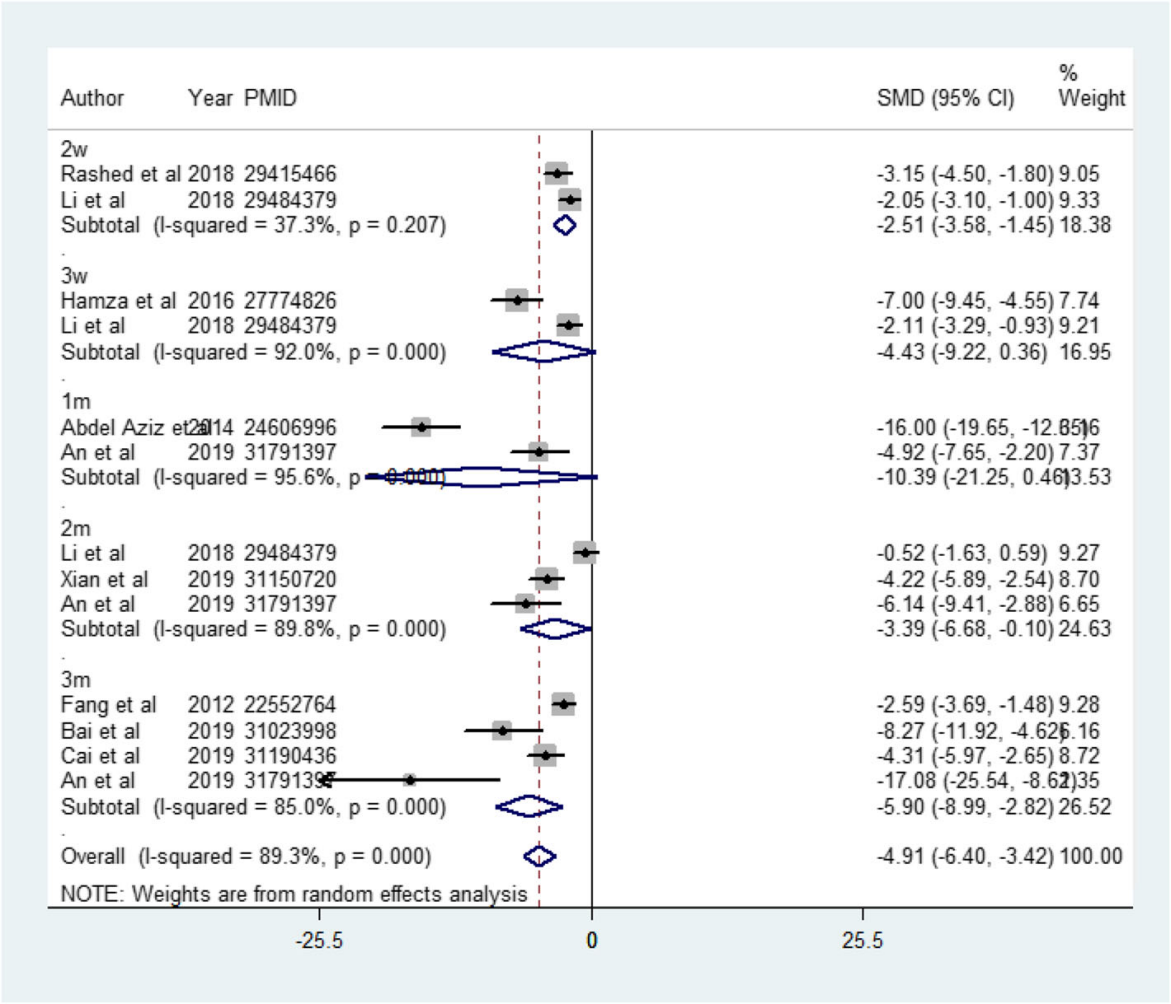
The effect of MSC treatment on blood urea nitrogen (BUN). 2w, 2 weeks; 3w, 3 weeks; 1m, 1 month; 2m, 2 months; 3m, 3 months

BUN was evaluated at 5 different time points, each of which was used by relatively few studies. At 2 weeks (2 studies included), 3 weeks (2 studies included), 1 month (2 studies included), 2 months (3 studies included), and 3 months (4 studies included), BUN decreased in the MSC-treated group, although no statistical significance was seen at 3 weeks or 1 month (2 weeks: SMD = − 2.514, 95%CI − 3.582 to − 1.447, *p* < 0.001; *I*^2^ = 37.3%; 3 weeks: SMD = − 4.432, 95%CI − 9.220 to − 0.356, *p* = 0.070; *I*^2^ = 92.0%; 1 month: SMD = − 10.392, 95%CI − 21.247 to 0.464, *p* = 0.061; *I*^2^ = 95.6%; 2 months: SMD = − 3.389, 95%CI − 6.679 to − 0.099, *p* = 0.044; *I*^2^ = 89.8%; 3 months: SMD = − 5.902, 95%CI − 8.988 to − 2.815, *p* < 0.001; *I*^2^ = 85.0%). The total effect on BUN was also analyzed, suggesting that MSCs decreased BUN (SMD = − 4.912, 95%CI − 6.402 to − 3.422, *p* < 0.001; *I*^2^ = 89.3%) (Fig. 3).

### Assessment of creatinine clearance rate

**Fig. 4:**
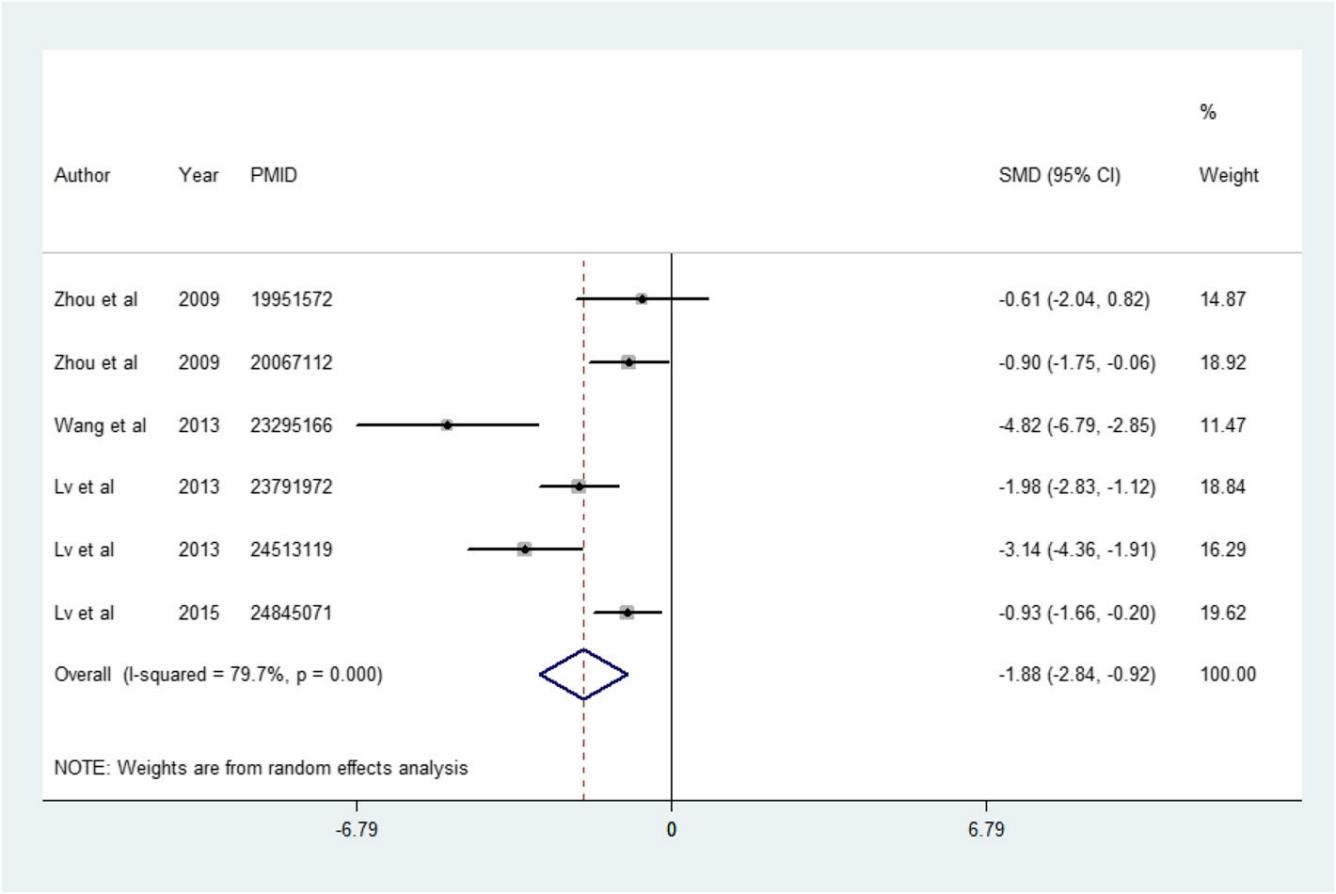
The effect of MSC treatment on a 2-month clearance of creatine rate (CCr)

The data of six studies were pooled to evaluate CCr at 2 months after MSC treatment; CCr was significantly decreased in the MSC-treated group compared to the DKD group (2 months: SMD = − 1.881, 95%CI − 2.842 to − 0.921, *p* < 0.001; *I*^2^ = 79.7%) (Fig. 4).

### Assessment of blood insulin level

Two studies assessed insulinemia. The insulin level increased at 3 months after MSC treatment, although the significance was not notable (3 months: SMD = 3.051, 95%CI − 0.091 to 6.193, *p* = 0.057; *I*^2^ = 90.3%).

### Assessment of urine protein

The measurement of urine protein varied in the included studies. Microalbuminuria, urinary albumin excretion, the urinary albumin/urinary creatinine ratio, and the urinary protein/creatinine ratio were used to assess urine protein excretion in the DKD animals.

Urinary albumin excretion levels at 1 month (2 studies included) and at 2 months (7 studies included) were observed to be lower in the MSC-treated group than in the DKD group, although no significance at 1 month was observed (1 month: SMD = − 6.507, 95%CI − 17.935 to 4.921, *p* = 0.264; *I*^2^ = 98.3%; 2 months: SMD = − 4.386, 95%CI − 5.891 to − 2.881, *p* < 0.001; *I*^2^ = 85.5%). The total effect on urinary albumin excretion was also analyzed, suggesting that MSCs decreased urinary albumin excretion (SMD = − 4.830, 95%CI − 6.602 to − 3.058, *p* < 0.001; *I*^2^ = 92.5%).

Microalbuminuria was detected at 3 weeks and 3 months; each of these time points was addressed by 2 studies that satisfied the inclusion criteria. Microalbuminuria was found to be decreased in the MSC-treated group at 3 months (3 weeks: SMD = − 9.112, 95%CI − 21.627 to 3.404, *p* = 0.154; *I*^2^ = 95.3%; 3 months: SMD = − 4.431, 95%CI − 5.771 to − 3.091, *p* < 0.001; *I*^2^ = 0.0%). The total effect on microalbuminuria was analyzed, suggesting that microalbuminuria was significantly lower in the MSC-treated group than in the DKD group (SMD = − 5.791, 95%CI − 8.681 to − 2.901, *p* < 0.001; *I*^2^ = 86.3%).

The urinary albumin/urinary creatinine ratios at 1 month (6 studies included) and at 2 months (10 studies included) were observed to be significantly lower in the MSC-treated group than in the untreated DKD group (1 month: SMD = − 2.419, 95%CI − 3.070 to − 1.769, *p* < 0.001; *I*^2^ = 0.0%; 2months: SMD = − 2.648, 95%CI − 3.454 to − 1.842, *p* < 0.001; *I*^2^ = 58.9%). The total effect on the urinary albumin/urinary creatinine ratio was analyzed, and the analysis suggested that the urinary albumin/urinary creatinine ratio was significantly lower in the MSC-treated group than in the DKD group (SMD = − 2.539, 95%CI − 3.075 to − 2.003, *p* < 0.001; *I*^2^ = 42.6%).

Urinary protein/creatinine ratios were not significantly different between the groups at 2 weeks (2 studies included) after MSC treatment (SMD = − 2.779, 95%CI − 7.617 to 2.059, *p* = 0.260; *I*^2^ = 92.6%).

### Assessment of kidney weight

Kidney weight and the kidney weight/body weight ratio were used to assess kidney hypertrophy. No significant intergroup difference in kidney weight was found between the MSC and untreated DKD groups at 1 month (2 studies included; SMD = − 0.674, 95%CI − 2.052 to 0.704, *p* = 0.337; *I*^2^ = 67.0%).

The kidney weight/body weight ratio was found to be significantly decreased in the MSC-treated group at 2 months (8 studies included, SMD = − 1.364, 95%CI − 2.164 to − 0.565, *p* = 0.001; *I*^2^ = 79.7%), while no significant difference was found between the two groups at 3 months (2 studies included, SMD = − 10.012, 95%CI − 29.753 to 9.729, *p* = 0.320; *I*^2^ = 97.0%). The total effect on the kidney weight/body weight ratio was analyzed, and the analysis suggested that a reduced kidney weight/body weight ratio was found in the MSC-treated group (SMD = − 1.624, 95%CI − 2.594 to − 0.655, *p* = 0.001; *I*^2^ = 86.9%).

### Assessment of body weight

There were 3 studies and 5 studies that assessed body weight at the 1-month and 2-month time points, respectively. No significant difference in 1-month body weight was found between the two groups (SMD = 2.634, 95%CI − 0.730 to 5.999, *p* = 0.125; *I*^2^ = 95.5%). At 2 months, the body weight of the MSC-treated groups significantly increased compared to that of the DKD groups (SMD = 0.903, 95%CI 0.346 to 1.459, *p* = 0.001; *I*^2^ = 40.2%). An overall effect of MSC treatment on body weight was also found (SMD = 1.499, 95%CI 0.461 to 2.536, *p* = 0.005; *I*^2^ = 87.3%).

### Assessment of renal fibrosis

Four included studies evaluated the percentage of glomerulosclerosis at 2 months after MSC treatment, and no significant difference was found (SMD = − 0.350, 95%CI − 4.173 to 3.473, *p* = 0.858; *I*^2^ = 96.2%).

Transforming growth factor-β (TGF-β) was measured at different time points using different methods. According to polymerase chain reaction (PCR) assays at 1 month (2 studies included) and 2 months (3 studies included) as well as western blot (WB) assays at 2 months (2 studies included), TGF-β was significantly decreased in the MSC-treated group (1-month PCR: SMD = − 3.281, 95%CI − 4.225 to − 2.337, *p* < 0.001; *I*^2^ = 4.2%; 2-month PCR: SMD = − 7.594, 95%CI − 13.274 to − 1.915, *p* = 0.009; *I*^2^ = 93.6%; 2-month WB: SMD = − 9.329, 95%CI − 11.569 to − 7.089, *p* < 0.001; *I*^2^ = 16.2%). The same was true for the total expression of TGF-β (SMD = − 6.839, 95%CI − 9.367 to − 4.312, *p* < 0.001; *I*^2^ = 90.5%).

Collagen I (Col-I) was detected by immunohistochemistry (IHC) and PCR. According to PCR at 2 months (3 studies included), Col-I was significantly decreased (SMD = − 11.856, 95%CI − 14.887 to − 8.826, *p* < 0.001, *I*^2^ = 41.3%) in the MSC-treated group, although no significant intergroup difference was found by IHC at 2 months (2 studies included; SMD = − 4.714, 95%CI − 10.670 to 1.242, *p* = 0.121; *I*^2^ = 95.3%). An overall effect on Col-I expression was also found (SMD = − 9.081, 95%CI − 14.233 to − 3.929, *p* = 0.001; *I*^2^ = 95.1%).

Three included studies evaluated fibronectin (FN) by IHC at 2 months after MSC treatment, and a statistically significant decrease was found in the MSC-treated group (SMD = − 7.781, 95%CI − 10.680 to − 4.881, *p* < 0.001; *I*^2^ = 71.3%).

Two studies evaluated α-smooth muscle actin (α-SMA) by WB at 1 month after MSC treatment, and 3 studies quantified its expression by PCR at 2 months. Both measures of α-SMA expression were significantly decreased in the MSC-treated group (1-month WB: SMD = − 2.514, 95%CI − 3.550 to − 1.479, *p* < 0.001; *I*^2^ = 0.0%; 2-month PCR: SMD = − 2.098, 95%CI − 3.721 to − 0.476, *p* = 0.011; *I*^2^ = 83.4%). An overall effect of MSC treatment on the expression of α-SMA was found (SMD = − 2.249, 95%CI − 3.311 to − 1.186, *p* < 0.001; *I*^2^ = 72.1%).

E-cadherin was quantified by WB at 1 month (2 studies included) after MSC treatment; the treatment was associated with a significant and notable decrease in E-cadherin deposition (SMD = 3.600, 95%CI 2.338 to 4.861, *p* < 0.001; *I*^2^ = 0.0%).

### Assessment of inflammatory mediators

Monocyte chemokine protein-1 (MCP-1) was detected by IHC at 2 months (2 studies included) after MSC treatment, and no significant difference was found between the two groups (SMD = − 8.913, 95%CI − 20.994 to 3.167, *p* = 0.148; *I*^2^ = 93.1%).

Tumor necrosis factor-α (TNF-α) was detected by enzyme-linked immunosorbent assay (ELISA) at 2 weeks (3 studies included) and by PCR at 1 month (2 studies included) after MSC treatment, both of which showed statistically significant decreases in the MSC-treated group (2-week ELISA: SMD = − 3.853, 95%CI − 7.207 to − 0.499, *p* = 0.024; *I*^2^ = 90.4%; 1-month PCR: SMD = − 4.369, 95%CI − 6.835 to − 1.903, *p* = 0.001; *I*^2^ = 57.5%). An overall effect of MSC treatment on the expression of TNF-α was found (SMD = − 4.027, 95%CI − 5.955 to − 2.098, *p* < 0.001; *I*^2^ = 84.9%).

### Risk of bias

**Fig. 5:**
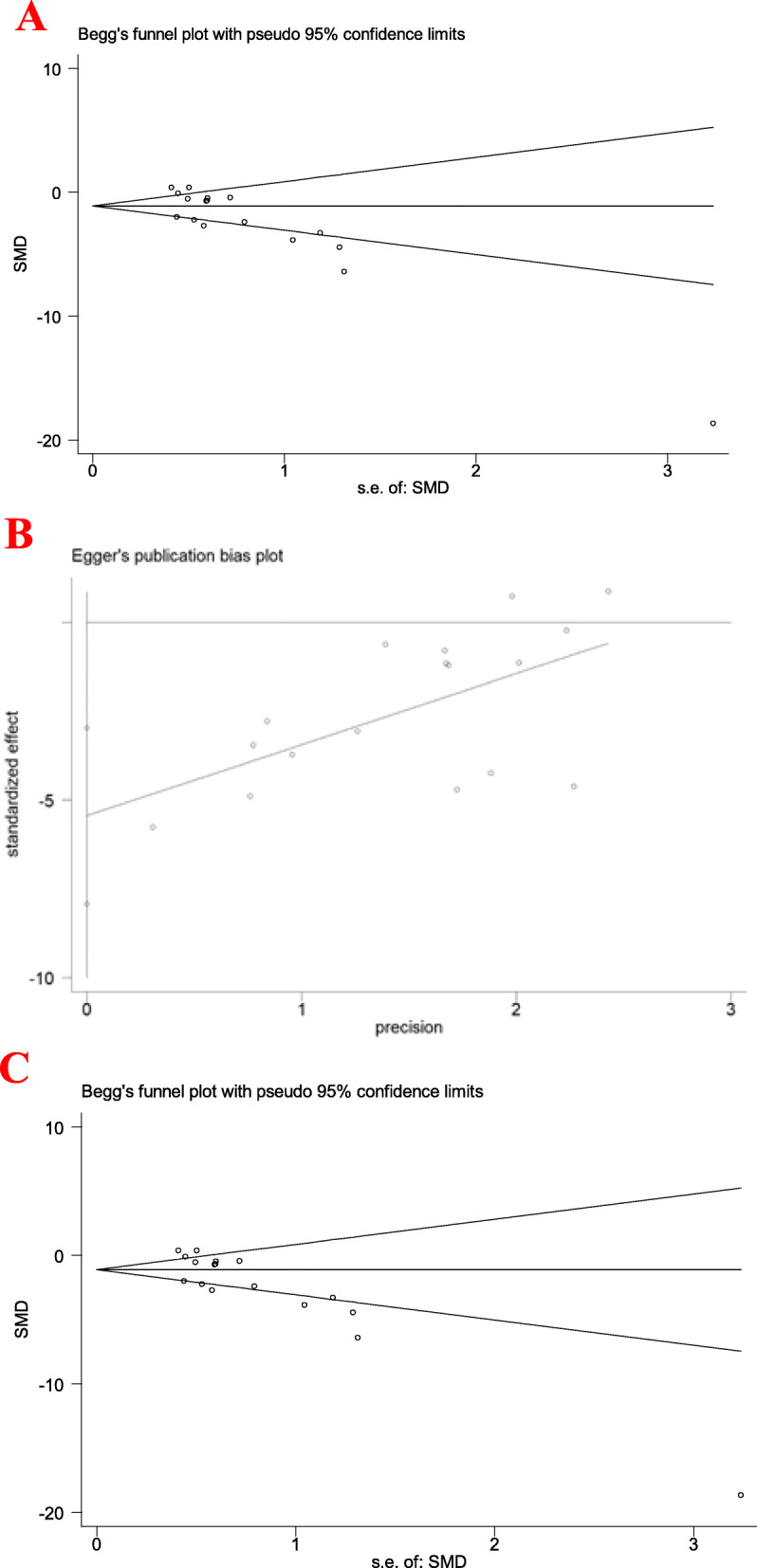
Publication bias. **a** Funnel plot. **b** Egger’s test. **c** Trim-and-fill method

Given sufficient data to assess publication bias, 2-month blood glucose was used for measurement. There was some degree of bias, indicated by a moderate asymmetry of the funnel plot, and Egger’s test showed *p* = 0.013. However, the trim-and-fill method did not identify any missing studies (Fig. 5).

## Discussion

Meta-analysis of medication in clinical trials is essential for clinical decisions in evidence-based medicine. Before medications are put into clinical use, preclinical experiments to explore their efficacy and safety must be performed, and these studies can be costly. In addition, in the absence of compelling evidence, testing directly on humans is both highly risky and unethical. A meta-analysis based on animals may provide a good reference to predict the outcomes of clinical trials. To evaluate the therapeutic effects of MSCs on DKD and review the mechanisms involved, we carried out this study. In this study, we performed a literature search with no species restrictions, yielding 32 animal studies in 28 publications and 1 clinical trial; on this basis, we conducted a meta-analysis of the animal studies and a systematic review.

The concept of DKD was proposed to replace DN in the Kidney Disease Outcomes Quality Initiative (K/DOQI) by the National Kidney Foundation (NKF) in 2007 and has been used to specify renal lesions caused by DM. DN is characterized by proteinuria ≥ 300 mg/day in a diabetic patient, with or without diabetic retinopathy and hypertension. However, with a new pathological classification of diabetic kidney lesions involving lesions of the tubules, interstitium, and/or vessels as determined by renal biopsy, the concept of DN has shifted to DKD in recent years focusing on clinical diagnosis. Because of the conceptual update, the precise distinction between DN and DKD was considered outside the scope of this study to avoid confusion, and both clinical entities were included.

In this paper, we found that MSCs might improve diabetic status, islet function, and glucose levels, as well as provide reno-protection. MSCs appeared to be effective in the treatment of diabetes, mitigating diabetic symptoms such as weight gain and decreased urine output and enhancing pancreatic islet function to improve inclusion secretion and glycemic control. Regarding the therapeutic effect of DKD, reductions in SCr, BUN, CCr, urinary protein, and renal hypertrophy were found in the MSC-treated group. In addition, molecular detection showed that MSCs might reduce the expression of renal fibrosis-related indicators, such as TGF-β, Col-I, FN, α-SMA, and E-cadherin, and the expression of inflammatory mediators such as MCP-1 and TNF-α.

To the best of our knowledge, this study is the first attempt to systemically evaluate MSC administration in DKD without species limitations. El-Badawy and El-Badri [42] conducted a meta-analysis of the therapeutic effects of different sources of stem cells in T1DM and T2DM by evaluating C-peptide, HbA1c, insulin requirements, and adverse effects, showing improved outcomes with stem cell therapy, especially CD34+ hematopoietic stem cell therapy. According to the study, the incidence of adverse effects was 21.72%, and no deaths were reported. To assess and quantify stem cells in animal studies of chronic kidney disease (CKD). Papazova et al. [43] performed a systematic review and meta-analysis and reported notable improvements in plasma creatinine, plasma urea, urinary protein, glomerular filtration rate (GFR), and blood pressure. Wang et al. [44] screened and pooled the data from small animal models of acute kidney injury (AKI) and CKD treated with MSCs and confirmed that impaired renal function was improved.

For glucose at 2 months, the moderate funnel plot asymmetry suggested the presence of bias, and Egger’s test showed *p* = 0.013; however, the trim-and-fill method did not show any missing studies. We detected significant heterogeneity, one of the inevitable drawbacks of animal meta-analyses; its causes may have included the following: different construction methods of animal models, different MSC treatment schemes, and different detection methods.

The therapeutic effects of MSC treatment seemed to be promising in animal studies, but the lone human investigation appeared to tell another story. That trial, a randomized, double-blind, placebo-controlled study of MSCs published in 2016, primarily assessed the safety over a 60-week follow-up and the efficacy over a 12-week follow-up. Regarding safety and tolerance, neither adverse events associated with MSCs nor persistent donor-specific anti-HLA antibodies were observed in the trial. However, except for interleukin-6 values and GFR stabilization, no significant difference from placebo was found in any other treatment outcome: urinary protein, CCr, lipid profile, HbA1c, blood pressure, TNF-α, adiponectin, TGF-β, uric acid, and fibroblast growth factor 23. Nevertheless, the results are not convincing, as they come from a single trial with a small sample size (*N* = 30).

Previous studies have indicated that MSCs can improve some other renal diseases. Chang et al. [45] assessed the effects of MSCs in an anti-Thy1.1-induced rat model of glomerulonephritis and found that intrarenal transplantation of MSCs with hypoxic preconditioning could reduce glomerular apoptosis, autophagy, and inflammation. Barbado et al. [46] conducted a clinical study in patients with lupus nephritis and found that MSC treatment dramatically improved proteinuria levels at the end of the first month, and the ameliorations were sustained throughout the follow-up period. Song et al. [47] conducted a study in rats with nephropathy induced by adriamycin (ADR) and showed that MSCs attenuated ADR-induced nephropathy by inhibiting NF-kB to diminish oxidative stress and inflammation and improve glomerulosclerosis and interstitial fibrosis.

How do MSCs improve renal lesions? MSC therapy has been reported to exert beneficial effects on renal impairment in animal models and patients [48–50]. However, the exact mechanisms of nephroprotection of MSCs remain unclear at present. To date, several potential mechanisms have been proposed. Immunoregulation is one important aspect, encompassing anti-inflammatory, antiapoptotic, and antioxidant action [51, 52]. In addition, one cannot ignore the inhibition of extracellular matrix accumulation, which may be achieved by promoting the secretion of antifibrotic factors and reducing the expression of renal fibrosis-related indicators [27, 53]. Protection of renal cells such as podocytes [51] and renal tubular epithelial cells [52] also deserves a place on the list. Proangiogenic potential is one of the functional characteristics of MSCs and may play a part in kidney repair [54]. Furthermore, attention should be paid to the homing of exogenously administered MSCs to specific parts or organs owing to the number of cells that come into play [15], and with the dedifferentiation of tubular cells into stem-like cells, there is a possibility of organ regeneration in AKI with MSC therapy [52].

In this study, a sensitivity analysis was performed, and we found that the results for sensitivity analysis were similar to those of non-sensitivity analyses. It might indicate that the results might be robust to some extent.

## Limitations

Only one clinical trial was included in this study, meaning that human data were seriously lacking. As for animal experiments, notable heterogeneity and bias left the conclusions uncertain. Because of the limited longevity of animals, the included animal experiments generally had short observation periods. Heterogeneity was observed in this meta-analysis due to the factors such as the experimental models of DM (e.g., animal species, method used to induce diabetes, and type of diabetes) and MSC treatment (e.g., source, dosage, frequency and route of administration, and timing of administration in relation to the onset of diabetic kidney disease). Sensitivity analysis should be performed by omitting each individual study. Concomitant effects of glycemia confound interpretations about direct therapeutic effects on renal injury. MSC-related adverse events were also limited. Overall poor quality of the experimental studies incorporated in the meta-analysis was found, and further attention should be paid to the design methodology as well as animal experiments with higher quality and larger samples in the future. If preclinical experiments yield sufficient evidence of efficacy and safety, it is expected that more human investigations will be conducted in the future.

## Conclusions

In animal models of DKD, MSCs might improve body weight, glycemic control, and pancreatic islet function to secrete insulin and reduce the SCr, BUN, CCr, urinary protein, and renal hypertrophy. MSCs can reduce the expression of inflammatory mediators and alleviate renal fibrosis. MSC therapy might be a potential treatment for DKD.

## Data Availability

Not applicable.

## Abbreviations

MSC: Mesenchymal stem cell
DKD: Diabetic kidney disease
NLM: National Library of Medicine
SYRCLE: Systematic Review Centre for Laboratory Animal Experimentation
SCr: Serum creatinine
BUN: Blood urea nitrogen
CCr: Creatinine clearance rate
UACR: Urinary albumin/urinary creatinine ratio
TGF-β: Transforming growth factor-β
Col-I: Collagen I
FN: Fibronectin
α-SMA: α-Smooth muscle actin
TNF-α: Tumor necrosis factor-α
PCR: Polymerase chain reaction
WB: Western blot
IHC: Immunohistochemical
ELISA: Enzyme-linked immunosorbent assay
GFR: Glomerular filtration rate
DM: Diabetes mellitus
DN: Diabetic nephropathy
RoB: Risk of bias
SMDs: Standard mean differences
OR: Odds ratio
CIs: Confidence intervals
STZ: Streptozotocin
BM-MSCs: Bone marrow mesenchymal stem cells
ADSCs: Adipose-derived stem cells
hUCB-MSCs: Human umbilical cord blood-derived mesenchymal stem cells
K/DOQI: Kidney Disease Outcomes Quality Initiative
NKF: National Kidney Foundation
CKD: Chronic kidney disease
